# Differential Expression of Multiple Disease-Related Protein Groups Induced by Valproic Acid in Human SH-SY5Y Neuroblastoma Cells

**DOI:** 10.3390/brainsci10080545

**Published:** 2020-08-12

**Authors:** Tsung-Ming Hu, Hsiang-Sheng Chung, Lieh-Yung Ping, Shih-Hsin Hsu, Hsin-Yao Tsai, Shaw-Ji Chen, Min-Chih Cheng

**Affiliations:** 1 Department of Psychiatry, Yuli Branch, Taipei Veterans General Hospital, Hualien 98142, Taiwan; hutsungming@mail2000.com.tw (T.-M.H.); pharm02@mail.vhyl.gov.tw (H.-S.C.); minipyng@gmail.com (L.-Y.P.); filvhsu@gmail.com (S.-H.H.); ashleytsai0808@gmail.com (H.-Y.T.); 2 Department of Future Studies and LOHAS Industry, Fo Guang University, Jiaosi, Yilan County 26247, Taiwan; 3 Department of Psychiatry, Mackay Medical College, New Taipei City 25245, Taiwan; shawjichen@gmail.com; 4 Department of Psychiatry, Taitung Mackay Memorial Hospital, Taitung County 95064, Taiwan

**Keywords:** valproic acid, proteomics, epigenetics, pathway analysis

## Abstract

Valproic acid (VPA) is a multifunctional medication used for the treatment of epilepsy, mania associated with bipolar disorder, and migraine. The pharmacological effects of VPA involve a variety of neurotransmitter and cell signaling systems, but the molecular mechanisms underlying its clinical efficacy is to date largely unknown. In this study, we used the isobaric tags for relative and absolute quantitation shotgun proteomic analysis to screen differentially expressed proteins in VPA-treated SH-SY5Y cells. We identified changes in the expression levels of multiple proteins involved in Alzheimer’s disease, Parkinson’s disease, chromatin remodeling, controlling gene expression via the vitamin D receptor, ribosome biogenesis, ubiquitin-mediated proteolysis, and the mitochondrial oxidative phosphorylation and electron transport chain. Our data indicate that VPA may modulate the differential expression of proteins involved in mitochondrial function and vitamin D receptor-mediated chromatin transcriptional regulation and proteins implicated in the pathogenesis of neurodegenerative diseases.

## 1. Introduction

Valproic acid (VPA), a branched-chain fatty acid, is widely used as an antiepileptic drug (AED) and an antimanic agent [1]. VPA is frequently used as an antimanic agent compared to lithium with a narrow therapeutic window and the strict limitations of using other medications that alter renal function, such as angiotensin-converting enzyme inhibitors or nonsteroidal anti-inflammatory drugs [2]. However, hepatic function and the blood level of VPA are monitored from starting use due to its side effects of liver or pancreas problems, such as loss of appetite, upper stomach pain, ongoing nausea or vomiting, dark urine, swelling in the face, or jaundice [3]. VPA also acts as a mood stabilizer in bipolar disorder and as a prophylactic for the prevention of migraine [4]. Besides, VPA is associated with anti-cancer activity [5,6]. Although VPA is generally safe to use and is broadly applicable, its mechanisms of action remain controversial. VPA can potentiate the inhibitory effect of γ-aminobutyric acid (GABA) transmission, possibly by enhancing GABA synthesis and blocking metabolism [7]. In addition, VPA may reduce voltage-gated sodium channel activity and modulate N-methyl-d-aspartate (NMDA) receptor function, effects that could explain its efficacy as both an antiepileptic drug and an antimanic agent [8].

There is growing evidence that the therapeutic effects of VPA involve the modulation of multiple protein signaling pathways. For example, microarray analysis of rat brain genes identified a variety of protein pathways modulated by VPA, including synaptic transmission, ion channels and transport, G-protein signaling, lipid, glucose, and amino acid metabolism, transcriptional and translational regulation, the phosphoinositol cycle, protein kinases and phosphatases, and apoptosis [9]. Du et al. reported in their study that the downregulation of transient receptor potential channel 1 (TRPC1) gene expression and function in neurons may be involved in the mood-stabilizing action of VPA [10], while Jiang et al. suggested that the downregulation of Homer1b/c is a potential mechanism for the neuroprotective effects of VPA and lithium [11].

Nevertheless, many of the detailed molecular mechanisms of VPA action are to date incompletely described or unknown. Comparative proteomics has emerged as a powerful tool in identifying differentially expressed proteins in biological systems under “experimental vs. control” or “disease vs. normal” conditions [12]. The identification of differentially expressed proteins can provide new insights into the mechanisms of biological processes such as development, cancer formation, and drug treatment effect. Isobaric tags for relative and absolute quantitation (iTRAQ) shotgun proteomic analysis is an isobaric labeling method used in quantitative proteomics by tandem mass spectrometry in order to identify multiple proteins from different functional classes in a single experiment [13]. The iTRAQ proteomic analysis is non-targeted and so allow for the unbiased investigation of numerous novel candidates across multiple biological pathways [14,15].

In the present study, we employed iTRAQ shotgun proteomic analysis, real-time quantitative PCR (RT-qPCR), and immunoblotting analysis to identify novel differentially expressed proteins in SH-SY5Y neuroblastoma cells treated with VPA. The SH-SY5Y neuroblastoma cells were derived from SK-N-SH cells, which were initially established from a bone marrow biopsy of a neuroblastoma patient [16]. The undifferentiated SH-SY5Y cells have been widely used as a model to study metabolism and neuroblastoma tumorigenesis [17,18]. Moreover, the undifferentiated SH-SY5Y cells have been used to study the effects of mood stabilizers and antipsychotic drugs, and Parkinson’s disease [19,20,21].

## 2. Materials and Methods

### 2.1. Cell Culture and VPA Treatment

The human SH-SY5Y neuroblastoma cell line (ATCC: CRL-2266) was cultured in Dulbecco’s modified Eagle’s medium (DMEM) supplemented with 10% fetal bovine serum (FBS), penicillin (100 units/mL), streptomycin (100 μg/mL), and L-glutamine (2 mM). The culture was maintained in a humidified atmosphere of 5% CO_2_ at 37 °C, and the medium was changed every two to three days. VPA sodium salt was purchased from Sigma-Aldrich (P4543). A stock solution was made in phosphate-buffered saline (PBS) and diluted in the medium to the final concentration indicated. An equal volume of PBS was added to control cultures.

### 2.2. Cell Viability Assay

The SH-SY5Y cells were plated in 96-well plates in a 10,000/well incubated for 24 h and then treated with VPA at the indicated concentrations for 24 h in serum-free DMEM. After VPA treatment, the cells were washed twice with PBS and cultured in DMEM with 10% FBS for two days. Subsequently, the cells were incubated with 0.5 mg/mL of the 3-(4,5-dimethyl-thiazol-2-yl)-2,5-diphenyl tetrazolium bromide (MTT) (Sigma Chemical Co., St. Louis, MO, USA) in DMEM for 4 h. Viable cells converted MTT to formazan, which is blue-purple when dissolved in dimethyl sulfoxide. Therefore, the color intensity (absorbance) is proportional to cell number. Absorbance at 545 nm was measured using a microplate reader (Varioskan Flash, Thermo Fisher Scientific, Vantaa, Finland) and % survival was calculated by dividing the absorbance of VPA-treated samples by absorbance of corresponding PBS-treated controls.

### 2.3. Protein Sample Preparation and iTRAQ Peptide Labeling

Cells treated as indicated were washed twice with cold PBS, re-suspended in lysis buffer containing 20 mM HEPES (pH = 7.6), 7.5 mM NaCl, 2.5 mM MgCl_2_, 0.1 mM EDTA, 0.1% TritonX-100, 0.1 mM Na_3_VO_4_, 50 mM NaF, and protease inhibitor cocktail (one mini tablet/10 mL, Roche Diagnostics GmbH, Mannheim, Germany). The homogenates were centrifuged at 13,000 rpm for 30 min at 4 °C, and the supernatants were stored at −80 °C until use.

Each specimen was taken out from −80 °C freezer, and the protein amount of cell lysate was determined using a Pierce BCA protein assay kit (Thermo Fisher Scientific, Rockford, IL, USA). The 4 protein samples of each group with an equal amount (20 μg per sample) were subjected to reduction (5 mM tris-(2-carboxyethyl)-phosphine, Sigma-Aldrich, Saint Louis, MO, USA), cysteine-blocking (10 mM methyl methanethiosulfonate, Sigma-Aldrich), and trypsin (0.8 μg, Promega) digestion at 37 °C for 16 h in a solution containing 200 mM triethylammonium bicarbonate buffer. The peptides were then labeled with iTRAQ reagent (Applied Biosystems, Foster City, CA, USA) according to the manufacturer’s protocol. After incubation at room temperature for 1 h, the 4 labeled peptide mixtures were pooled, dried by vacuum centrifugation, and stored at −80 °C until use.

### 2.4. LC-MS/MS Analysis

The dried peptide mixtures were reconstituted and desalted using a homemade C18 microcolumn. The resulted peptides were reconstituted and loaded onto a homemade column (Luna SCX 5 μm, 0.5 × 120 mm) at a flow rate of 3 μL/min for 40 min. The peptides were then eluted fractionated to 22 fractions by eluting with 0 to 100% HPLC buffer B (0.5 M ammonium chloride/30% acetonitrile/0.1% formic acid) using on-line 2D-HPLC (Dionex Ultimate 3000, Thermo Fisher, San Jose, CA, USA). Each SCX fraction was diluted in-line before trap onto a reverse-phase column (Zorbax 300SB-C18, 0.3 × 5 mm; Agilent Technologies, Santa Clara, CA, USA). The peptides were then separated on a homemade column (HydroRP 2.5 μm, 75 μm I.D. × 24 cm with a 15 μm tip) using a multi-step gradient of HPLC buffer C (99.9% acetonitrile/0.1% formic acid) for 65 min with a flow rate of 0.23 μL/min. The LC apparatus was coupled with a 2D linear ion trap mass spectrometer (LTQ-Orbitrap ELITE; Thermo Fisher, San Jose, CA, USA) operated using Xcalibur 2.2 software (Thermo Fisher, San Jose, CA, USA). The full-scan MS was performed in the Orbitrap over a range of 400 to 1600 Da and a resolution of 120,000 at *m*/*z* 400. For proteome analysis, the 12 data-dependent MS/MS scan events (HCD) were followed by one MS scan for the 12 most abundant precursor ions in the preview MS scan. The *m*/*z* values selected for MS/MS were dynamically excluded for 80 s with a relative mass window of 20 ppm. The electrospray voltage was set to 2.0 kV, and the temperature of the capillary was set to 200 °C. MS and MS^n^ automatic gain control were set to 1000 ms (full scan), 300 ms (MS^2^ for HCD), or 3 × 10^6^ ions (full scan), and 3 × 10^4^ ions (MS^2^ for HCD) for maximum accumulated time or ions, respectively. 

### 2.5. Protein Identification

Liquid chromatography-tandem mass spectrometry (LC-MS/MS) data were analyzed using Proteome Discoverer software (version 1.4, Thermo Fisher Scientific), including the reporter ions quantifier node for iTRAQ quantification. The MS/MS spectra were searched against the UniProt sequence database (released on 16 March 2016, extracted for *Homo sapiens*, 20,199 entries) using the Mascot search engine (Matrix Science, London, UK; version 2.5). For peptide identification, 10 ppm of mass tolerance was permitted for intact peptide masses and 0.05 Da for HCD fragment ions with allowance for two missed cleavages made by trypsin digestion: oxidized methionine; acetyl (protein N-terminal); iTRAQ4plex (N-terminal); and iTRAQ4plex (lysine), as variable modifications and methylthio (cysteine) as the fixed modification. The protein ratio was calculated using the intensity of iTRAQ reporter ions (114, 115, 116, and 117) in the spectrum, which matched to the specific peptide in the sequence database. If one of the 4 channels was missing and the missing value was not replaced (e.g., replaced to 0 or minimal intensity of reporter ion in the LC-MS/MS experiment), the protein ratio would be left to blank. The false discovery rate of peptide-spectrum matching was calculated using Percolator (*q* value < 0.01) [22]. Protein ratio with at least two peptide identifications was normalized based on the median ratio of all proteins in each group. After ratio normalization, the mean ± 1.5 × standard deviation of the log2 transformed ratio was used as thresholds for differential protein selection.

### 2.6. Pathway Analysis

DAVID (the database for annotation, visualization and integrated discovery) is a web-based bioinformatics resource intended for functional interpretation of large lists of genes or proteins [23]. In total, 1195 differentially expressed proteins (mean ± 1.5 × standard deviation (SD) of the log2) were subjected to the DAVID database using the official gene symbol method (DAVID v6.8) [24]. Differentially expressed proteins associated with the Biological Biochemical Image Database (BBID), Biocarta, and Kyoto Encyclopedia of Genes and Genomes (KEGG) pathways were highlighted, annotated, and viewed in pathway maps [25,26,27].

### 2.7. Immunoblotting Analysis

Cellular protein was mixed with sample buffer (62.5 mM Tris-HCl pH = 6.8, 2% SDS, 25% glycerol, 0.01% Bromophenol Blue, 5% β-mercaptoethanol) and denatured by heating at 95 °C for 10 min. Immunoblotting analysis was performed using standard protocols with the following primary antibodies: rabbit anti-ATP5D (A9929, ABclonal, Woburn, MA, USA); rabbit anti-ATP5J (tcea21587, Taiclone, Taipei, Taiwan); rabbit anti-DPH6 (23993-1-AP, Proteintech, Rosemont, IL, USA); rabbit anti-FBXO4 (tcea12933, Taiclone); rabbit anti-FSTL1 (A15789, ABclonal); mouse anti-glyceraldehyde 3-phosphate dehydrogenase (GAPDH) (G8795, Sigma-Aldrich); mouse anti-HDAC1 (GTX100513, GeneTex, Hsinchu City, Taiwan); rabbit anti-HDAC2 (GTX112957, GeneTex); rabbit anti-HIST1H2B (MAB15119, Abnova, Taipei, Taiwan); rabbit anti-KCNAB2 (17890-1-AP, Proteintech); rabbit anti-NPTX2 (A12031, ABclonal); rabbit anti-SCG3 (A7799, ABclonal); rabbit anti-SMARCA4 (tcea559, Taiclone); rabbit anti-UBE1L (tcea10708, Taiclone); rabbit anti-UBE2D1 (tcea7105, Taiclone); rabbit anti-UQCRB (tcea18400, Taiclone); rabbit anti-VDAC1 (tcea521, Taiclone); rabbit anti-VSNL1 (A6999, ABclonal). Horseradish peroxidase-conjugated donkey anti-rabbit IgG (NA934, Amersham, Little Chalfont, UK) or goat anti-mouse IgG (074-186, KPL) were used as the secondary antibodies. Immunolabeled proteins were visualized using the ECL detection system (GE Health Care Bio-Sciences AB, Uppsala, Sweden). The membrane was then stained by submersion in Amido Black (0.1% [*w*/*v*] Naphthol blue-black in 10% methanol, 2% acetic acid, Sigma-Aldrich, Saint Louis, MO, USA) for the control of protein loading and transfer efficiency. Immunoblot intensity was assessed using NIH ImageJ software (NIH image) [28]. Treatment group means were compared by analysis of variance (ANOVA). A *p* < 0.05 was considered significant for all tests. 

### 2.8. Total RNA Preparation and RT-qPCR

Total RNA of cells was purified using TRIzol reagent (Invitrogen) according to the manufacturer’s standard protocol and stored at −80 °C until use. cDNA was prepared using SuperScript^TM^ III Reverse Transcriptase (Invitrogen) according to the manufacturer’s standard protocol. The RT-qPCR was performed using an Applied Biosystems PRISM7900 Sequence Detection System. We used a comparative ^ΔΔ^Ct method to validate the differential gene expression. The ^ΔΔ^Ct value of each sample was obtained by subtracting the Ct of the target gene from the Ct of the *GAPDH* gene of the sample as the endogenous gene for normalization and then from the ^Δ^Ct of the first sample. Differences of fold change (2^^−ΔΔ^CT) for selected genes among four groups were assessed by ANOVA test. All data were expressed as mean ± SD. Statistically significant differences were defined as those with a *p*-value <0.05. All experiments were performed in at least triplicate.

## 3. Results

### 3.1. Effects of VPA on SH-SY5Y Cell Viability

**Figure 1:**
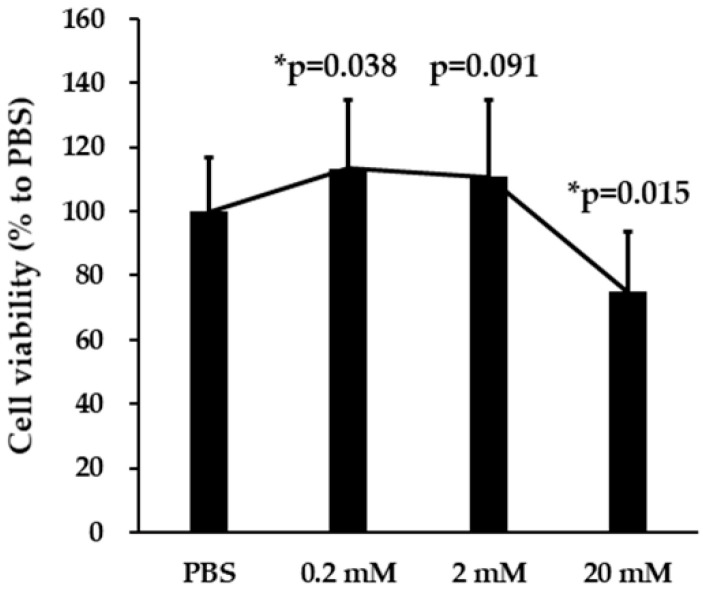
Valproic acid (VPA) has reciprocal concentration-dependent effects on SH-SY5Y cell viability. The 3-(4,5-dimethyl-thiazol-2-yl)-2,5-diphenyl tetrazolium bromide (MTT) assay was used to estimate cell number following 24 h treatment with the indicated VPA concentration. The results were calculated as the 545 nm optical density (OD545) of VPA-treated cultures relative to phosphate-buffered saline (PBS)-treated control cultures and expressed as means ± SD. VPA enhanced survival or proliferation at the low concentration and reduced survival or proliferation at the high concentration. A statistically significant difference between VPA- and PBS-treated cultures was detected by analysis of variance (ANOVA), and a post hoc test using the least significant difference (LSD) setting was performed (* *p* < 0.05, n = 24).

The effects of VPA (0.2, 2, and 20 mM for 24 h) on SH-SY5Y cell viability were examined using the MTT assay (Figure 1). The % survival relative to PBS-treated controls was 75% after exposure to 20 mM VPA for 24 h (*p* = 0.015) and 113% after exposure to 0.2 mM VPA (*p* = 0.038). Therefore, high-dose VPA had modest inhibitory effects on cell survival and/or proliferation, while low-dose VPA appeared to promote survival and/or proliferation.

### 3.2. Identification of Differentially Expressed Proteins by the iTRAQ Analysis

The iTRAQ shotgun proteomic analysis method was used to identify proteins differentially expressed by SH-SY5Y cells exposed to different VPA concentrations for 24 h. Among 5325 protein identifications in the iTRAQ-labeled samples, 5288 proteins were able to be quantified in all groups (Table 1). Global normalization [29] was used to correct the starting protein amount in different treatments. By assuming the most proteins in different treatments remained unchanged, all protein ratios were normalized using the median ratio in each group. Protein ratio with at least two peptide identifications was normalized based on the median ratio of all proteins in each group (Tables S1 and S2). After ratio normalization, mean ± 1.5 × SD of log2 transformed ratio was used as thresholds for differential protein selection. After global normalization, a total of 1195 differentially expressed proteins (mean ± 1.5 × SD) were subjected to pathway enrichment analysis. These differentially expressed proteins are listed in the Supplementary Table S3.

**Figure 2:**
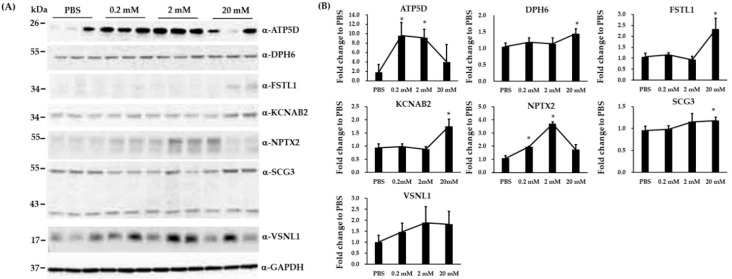
Immunoblotting analysis to validate the differential expression for 7 proteins in VPA-treated and PBS-treated biological replicated SH-SY5Y cells. (**A**) Immunoblotting showed the expression of ATP5D, DPH6, FSTL1, KCNAB2, NPTX2, SCG3, and VSNL1 in VPA-treated and PBS-treated SH-SY5Y cells. (**B**) Quantification showing the fold differences in the expression of ATP5D, DPH6, FSTL1, KCNAB2, NPTX2, SCG3, and VSNL1 between VPA-treated groups and PBS control. Glyceraldehyde 3-phosphate dehydrogenase (GAPDH) was a loading control. Data are expressed as fold change to PBS ± SD (* *p* < 0.05, n = 3).

Because we were not able to validate all the differentially expressed proteins identified from iTRAQ assay, 2 down-expressed proteins (KRT6A and KRT14) and 7 up-expressed proteins (ATP5D, DPH6, FSTL1, KCNAB2, NPTX2, SCG3, and VSNL1) in all treated groups were selected for verification with biological replicated SH-SY5Y cells using the immunoblotting assay. However, KRT6A and KRT14 protein were not well detected in the SH-SY5Y cells (data not shown). We compared the expression levels of seven up-expressed proteins in biological replicated SH-SY5Y cells treated with either PBS or VPA (0.2, 2, and 20 mM) using immunoblotting analysis. The fold differences in the expression of these proteins between VPA-treated groups and PBS control were calculated (Figure 2A,B). We were able to verify 6 proteins (ATP5D, DPH6, FSTL1, KCNAB2, NPTX2, and SCG3) that were significantly up expressed by the treatment of VPA.

### 3.3. Pathway Enrichment Analysis

Next, the proteins with altered expression in each group were submitted to a pathway analysis using the functional annotation of DAVID 6.8. The BBID, Biocarta, and KEGG pathway analyses showed that differentially expressed proteins in the different VPA concentrations associated with several pathways and diseases (Supplementary Table S4). After Benjamini multiple correlations (*p* < 0.05), many differentially expressed proteins were found to be involved in the control of gene expression via the vitamin D receptor, oxidative phosphorylation, electron transport in mitochondria, ribosome biogenesis in eukaryotes, Parkinson’s disease, ubiquitin-mediated proteolysis, and Alzheimer’s disease (Table 2). VPA altered the expression of 11 proteins (ATP5A1, ATP5B, ATP5D, ATP5F1, ATP5J, SDHA, SDHB, UQCRB, UQCRC1, UQCRC2, and UQCRH) involved in Alzheimer’s disease (Figure S1A); 16 proteins (ATP5A1, ATP5B, ATP5D, ATP5F1, ATP5J, SDHA, SDHB, SLC25A6, UBA7, UQCRB, UQCRC1, UQCRC2, UQCRFS1, UQCRH, VDAC1, and VDAC3) involved in Parkinson’s disease (Figure S1B); 13 proteins (ATP5A1, ATP5B, ATP5D, ATP5F1, ATP5J, ATP5L, ATP6V1G1, SDHA, SDHB, UQCRB, UQCRC1, UQCRC2, and UQCRH) involved in oxidative phosphorylation (Figure S1C); 5 proteins (ATP5A1, SDHA, SDHB, SLC25A6, and UQCRC1) involved in electron transport reaction in mitochondria (Electron Transprot Chain) [30]; 9 proteins (BAZ1B, HDAC1, NCOA2, SMARCA4, SMARCC1, SMARCD1, SMARCE1, SUPT16H, and TOP2B) involved in the control of gene expression by vitamin D receptor [31]; 10 proteins (ANAPC4, CDC23, FBXO2, FBXO4, NEDD4, TRAF6, UBE2D1, UBE2E1, UBE2E3, and UBE2G2) involved in ubiquitin mediated proteolysis (Figure S1D); 10 proteins (BMS1, DKC1, GNL2, GTPBP4, LSG1, NOP10, RIOK2, TCOF1, WDR36, and WDR75) involved in ribosome biogenesis in eukaryotes (Figure S1E).

### 3.4. Validation of Three Differentially Expressed Epigenetic Proteins by Immunoblotting

**Figure 3:**
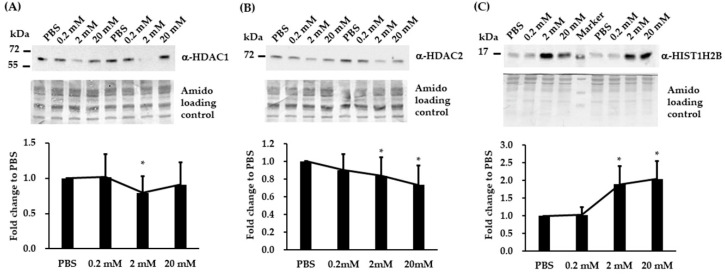
VPA induces the differential expression of the epigenetic regulators HDAC1, HDAC2, and HIST1H2B in SH-SY5Y cells. (**A**–**C**) Representative immunoblots and quantification of target protein band intensities for lysates from SH-SY5Y cultures treated with the indicated VPA concentration or PBS (control). (**A**) HDAC1. (**B**) HDAC2. (**C**) HIST1H2B. The results are expressed as means ± SD. A statistically significant difference between VPA and PBS was detected by ANOVA (* *p* < 0.05, n = 5 to 10).

Because VPA is an inhibitor of histone deacetylases (HDACs), we selectively performed immunoblotting analysis on three epigenetic proteins (HDAC1, HDAC2, and HIST1H2B) identified by iTRAQ assay. Immunoblotting analysis revealed that VPA reduced HDAC1 expression at 2 mM (Figure 3A) and HDAC2 at 2 and 20 mM (Figure 3B), while VPA upregulated HIST1H2B expression at 2 and 20 mM (Figure 3C).

### 3.5. Immunoblotting and RT-qPCR Analysis in Biological Replicated SH-SY5Y Cells 

**Figure 4:**
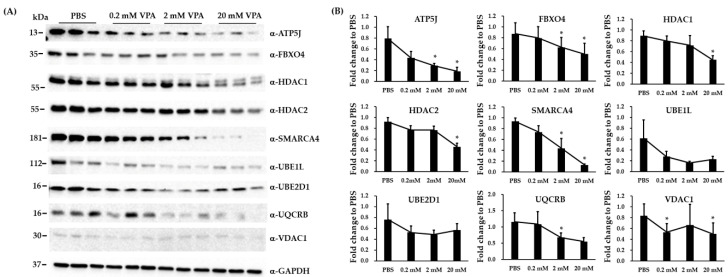
Immunoblotting analysis to validate the differential expression for 9 proteins in VPA-treated and PBS-treated biological replicated SH-SY5Y cells. (**A**) Immunoblotting showed the expression of ATP5J, FBXO4, HDAC1, HDAC2, SMARCA4, UBE1L, UBE2D1, UQCRB, and VDAC17 in VPA-treated and PBS-treated SH-SY5Y cells. (**B**) Quantification showing the fold differences in the expression of ATP5J, FBXO4, HDAC1, HDAC2, SMARCA4, UBE2D1, UQCRB, and VDAC1 between VPA-treated groups and PBS control. GAPDH was a loading control. The data are expressed as fold change to PBS ± SD (* *p* < 0.05, n = 3 to 6).

**Figure 5:**
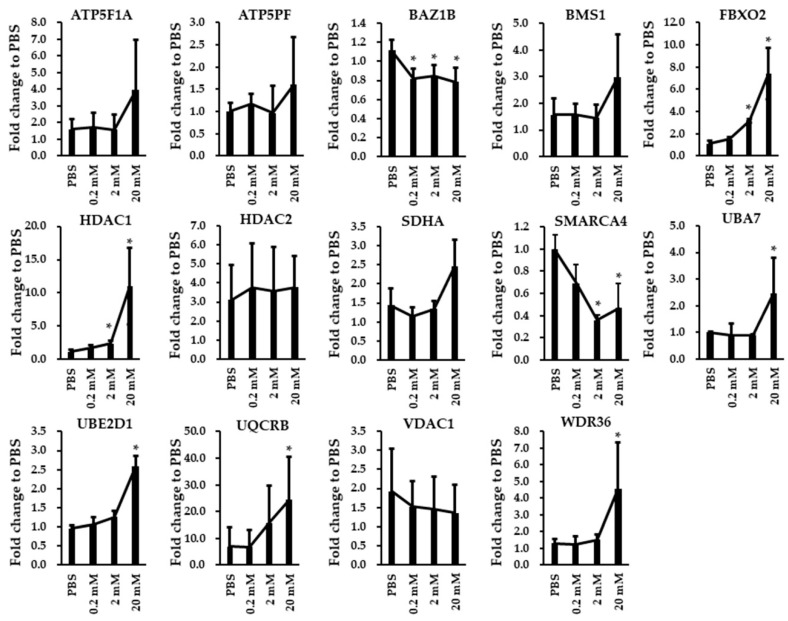
RT-qPCR assay showing the expression of 14 genes (*ATP5F1A*, *ATP5PF*, *BAZ1B*, *BMS1*, *FBXO2*, *HDAC1*, *HDAC2*, *SDHA*, *SMARCA4*, *UBA7*, *UBE2D1*, *UQCRB*, *VDAC1*, and *WDR36*) in VPA-treated and PBS-treated SH-SY5Y cells. The *GAPDH* gene was used as the endogenous gene for normalization. The data are expressed as fold change to PBS ± SD (* *p* < 0.05, n = 3 to 6).

Nine differentially expressed proteins and 14 differentially expressed genes on enriched pathways were selected for verification with biological replicated SH-SY5Y cells. We compared the expression levels of nine proteins in biological replicated SH-SY5Y cells treated with either PBS or VPA (0.2, 2, and 20 mM) using immunoblotting analysis. The fold differences in the expression of these proteins between VPA-treated groups and PBS control were calculated (Figure 4A,B). Seven proteins (ATP5J, FBXO4, HDAC1, HDAC2, SMARCA4, UQCRB, and VDAC1) were differentially expressed by the treatment of VPA (Figure 4B). We compared the mRNA expression levels of 14 genes in biological replicated SH-SY5Y cells treated with either PBS or VPA (0.2, 2, and 20 mM) using RT-qPCR assay. The fold differences between VPA-treated groups and PBS control of these 14 genes are shown in Figure 5. Primer sequences and the size of each amplicon are listed in the Supplementary Table S5. Among the 14 genes assayed, we found that eight genes were differentially expressed by the treatment of VPA, including *BAZ1B*, *FBXO2*, *HDAC1*, *SMARCA4*, *UBA7*, *UBE2D1*, *UQCRB*, and *WDR36*. In the *HDAC1*, *UBE2D1*, *UQCRB*, and *WDR36* gene, change in mRNA levels and protein levels were opposite by the treatment of VPA.

## 4. Discussion

VPA is an inhibitor of histone deacetylases (HDACs) and has pleiotropic effects on many biological processes such as cancer cell growth arrest, proliferation, differentiation, invasion, apoptosis, immune system, and neuroprotection [32,33,34,35,36,37]. Several studies indicate that HDAC inhibitors may be promising therapeutic agents for neurodegenerative diseases [38,39]. Recently, VPA emerges as a novel potent anti-cancer drug potentially applicable for clinical practice [40]. However, the comprehensive mechanisms underlying VPA anti-cancer effects and neuroprotection remain to be elucidated due to its pleiotropic effects on multiple signaling pathways. In line with the findings from the present study and other research groups [41,42], we used proteomic shotgun analysis to identify multiple differentially expressed proteins in VPA-treated SH-SY5Y neuroblastoma cells. Among these differentially expressed proteins, we were able to confirm six proteins that are differentially up expressed under the administration of VPA. However, two down-expressed proteins (KRT6A and KRT14) were not well detected in the SH-SY5Y cells. The results may be due to the antibody specificity. According to subsequent pathway analyses, VPA appears to regulate a myriad of physiological and two neurodegenerative disease-associated signaling pathways, including Parkinson’s disease and Alzheimer’s disease. Immunoblotting and RT-qPCR confirmed that VPA regulated a subset of proteins and genes enriched in these pathways. However, in the *HDAC1*, *UBE2D1*, *UQCRB*, and *WDR36* gene, change in mRNA levels and protein levels were opposite in SH-SY5Y cells treated with VPA. The lack of correlation between mRNA and protein abundances can be explained by transcriptional regulation, translation efficiency, and protein turnover rates [43,44]. 

In the current study, VPA altered the expression of 11 proteins implicated in Alzheimer’s disease (ATP5A1, ATP5B, ATP5D, ATP5F1, ATP5J, SDHA, SDHB, UQCRB, UQCRC1, UQCRC2, and UQCRH), while 16 proteins implicated in Parkinson’s disease (ATP5A1, ATP5B, ATP5D, ATP5F1, ATP5J, SDHA, SDHB, SLC25A6, UBA7, UQCRB, UQCRC1, UQCRC2, UQCRFS1, UQCRH, VDAC1, and VDAC3). Of note, except UBA7, above all differentially expressed proteins are implicated in mitochondrial oxidative phosphorylation chain. Furthermore, our findings identified targeting mitochondrion proteins as an approach to alleviate Alzheimer’s disease and Parkinson’s disease. Alzheimer’s disease is a complex neurodegenerative disease with multifaceted pathogenesis that usually starts slowly and gradually worsens over time [45]. Both in vitro and in vivo studies have shown strong modulatory actions of VPA on neurogenesis, neurite outgrowth, and neuronal survival [19,46]. VPA promoted neurogenesis and GSK-3β dependent neurite outgrowth in a mouse model of Alzheimer’s disease [47,48]. Therefore, VPA may be useful for the treatment of behavioral disturbance in dementia patients [49,50,51,52]. Parkinson’s disease is a progressive neurodegenerative disorder that primarily affects the motor control system. Animal model studies support the therapeutic potential of VPA may be Parkinson’s disease [53]. Clinically, VPA is used to treat dopamine dysregulation syndrome in Parkinson’s disease [54], although the molecular mechanisms underlying this therapeutic effect are to date unclear. 

Interestingly, we found that VPA altered the expression levels of 15 mitochondrial proteins involved in oxidative phosphorylation (ATP5A1, ATP5B, ATP5D, ATP5F1, ATP5J, ATP5L, ATP6V1G1, SDHA, SDHB, UQCRB, UQCRC1, UQCRC2, and UQCRH) and five involved in mitochondrial electron transport chain (ATP5A1, SDHA, SDHB, SLC25A6, and UQCRC1). The mitochondrion is a double-membrane-bound organelle and produces ATP from products of the citric acid cycle, fatty acid oxidation, and amino acid oxidation. In eukaryotes, electron transport reaction is found in the inner mitochondrial membrane, where it serves as the site of oxidative phosphorylation. Mitochondrion dysfunction has been suggested as a contributory factor in the pathology of various neuropsychiatric disorders, including neurodegenerative disorders and neurodevelopmental disorders [55,56,57]. Taken together, the clinical efficacy of VPA may be related to mitochondrial electron transport or oxidative phosphorylation chain in patients with neurodegenerative disease.

In the current study, VPA altered the expression of nine proteins involved in the control of gene expression by vitamin D receptor (BAZ1B, HDAC1, NCOA2, SMARCA4, SMARCC1, SMARCD1, SMARCE1, SUPT16H, and TOP2B). Notably, SMARCA4, SMARCC1, SMARCD1, and SMARCE1 are members of the SWI/SNF family of proteins, whose members display helicase and ATPase activities and which are thought to regulate transcription of specific genes by altering the chromatin structure around those genes [58]. VPA is an inhibitor of HDACs, which together with histone acetyltransferases control the state of histone acetylation, suggesting that the epigenetic regulation of gene expression may contribute to effects of VPA on neuropsychiatric diseases [33,59]. In the present study, the immunoblotting assay confirmed that VPA reduced HDAC1 and HDAC2 expression and induced HIST1H2B expression. The results suggest that the inhibition of HDACs and the concomitant enhancement of histone acetylation (which facilitates gene transcription) contribute to the clinical efficacy of VPA. Vitamin D is essential for bone health, and vitamin D deficiency is linked to osteoporosis and reduced mineral density [60]. Clinical observations have shown that chronic VPA use has variable effects on bone metabolism. For example, the long-term use of AEDs, including VPA, has been associated with decreased bone mineral density [61], including in young epilepsy patients [62]. However, the mechanisms for this potential side effect are unclear. A previous iTRAQ proteomic study reported that VPA reduces collagen and osteonectin in a cellular model of spinal muscular atrophy [63]. Vitamin D receptor regulates chromatin structure in cooperation with various histone modifiers and chromatin remodelers [64,65]. Taken together, we speculate that VPA actions on bone metabolism are associated with vitamin D receptor-mediated chromatin transcriptional regulation of target genes.

In addition, we found that VPA can modulate the expression of proteins associated with ubiquitin-mediated proteolysis and ribosome biogenesis, which is in accordance with whole-genome microarray analysis of primary tumors from VPA-treated patients showing upregulation of several hundred genes, including genes that are related to ribosomal proteins and ubiquitin-mediated proteolysis [66]. 

In the present study, we observed that VPA could modulate the differential expression of several proteins in SH-SY5Y cells by iTRAQ shotgun proteomic analysis. However, this study has several limitations. Firstly, we did not perform a biological replicate in the iTRAQ experiment. Thus, we cannot account for biological variations among different cell passages. Secondly, the sample size is small and was conducted using only the SH-SY5Y cell line. Thirdly, differential protein expression was confirmed by immunoblotting and RT-qPCR assay for only a small subset of proteins. Hence, to obtain more insight into the molecular mechanism of VPA, we need to verify other genes and proteins in the future, and further replicated studies are needed to consolidate the findings in this study.

## 5. Conclusions

In conclusion, by using iTRAQ analysis, immunoblotting, and RT-qPCR analysis, we observed that VPA could induce the differential expression of several proteins in SH-SY5Y cells. The identification of these differentially expressed proteins both enhances our knowledge of the molecular mechanisms underlying the clinical effects of VPA, such as the ubiquitin-mediated proteolysis, ribosome biogenesis in eukaryotes, chromatin-mediated transcriptional regulation, mitochondria electron transport reaction, and mitochondria oxidative phosphorylation. It provides potentially novel pathogenic mechanisms and treatment targets for various neurodegenerative diseases and cancers.

## Figures and Tables

**Table 1:** Summary of quantification results.

# Proteins	0.2 mM VPA/PBS	2 mM VPA/PBS	20 mM VPA/PBS
Total Quantification	5293	5295	5294
≥mean + 1.5 × s.d. (fold change)	213 (≥1.35)	261 (≥1.28)	302 (≥1.39)
≤mean − 1.5 × s.d. (fold change)	229 (≤0.73)	332 (≤0.76)	280 (≤0.72)
≥1.5 fold change	83	49	176
≤1.5 fold change	122	117	153

**Table 2:** Pathway enrichment.

Group	Trend	Category	Term	Count	Proteins
0.2 mM/PBS	Down	KEGG_pathway: map04120	Ubiquitin mediated proteolysis	10	UBE2E3, NEDD4, UBE2G2, ANAPC4, FBXO2, CDC23, FBXO4, TRAF6, UBE2D1, UBE2E1
2 mM/PBS	Up	KEGG_pathway: map05012	Parkinson’s disease	10	UQCRC2, ATP5D, UQCRH, ATP5F1, UBA7, UQCRFS1, VDAC3, VDAC1, ATP5J, UQCRB
2 mM/PBS	Down	BIOCARTA	Control of Gene Expression by Vitamin D Receptor ^#^	9	NCOA2, BAZ1B, SMARCE1, HDAC1, SMARCC1, SMARCD1, SUPT16H, TOP2B, SMARCA4
2 mM/PBS	Down	KEGG_pathway: map03008	Ribosome biogenesis in eukaryotes	10	WDR75, GTPBP4, WDR36, DKC1, TCOF1, LSG1, GNL2, RIOK2, BMS1, NOP10
20 mM/PBS	Up	BIOCARTA	Electron Transport Reaction in Mitochondria ^$^	5	SDHA, SDHB, UQCRC1, SLC25A6, ATP5A1
20 mM/PBS	Up	KEGG_pathway: map00190	Oxidative phosphorylation	13	ATP5D, UQCRC2, UQCRC1, ATP5B, ATP5F1, ATP6V1G1, SDHA, SDHB, UQCRH, ATP5L, ATP5A1, UQCRB, ATP5J
20 mM/PBS	Up	KEGG_pathway: map05012	Parkinson’s disease	12	UQCRC2, SDHA, ATP5D, SDHB, UQCRC1, UQCRH, ATP5B, SLC25A6, ATP5F1, ATP5A1, ATP5J, UQCRB
20 mM/PBS	Up	KEGG_pathway: map05010	Alzheimer’s disease	11	UQCRC2, SDHA, ATP5D, SDHB, UQCRC1, UQCRH, ATP5B, ATP5F1, ATP5A1, ATP5J, UQCRB

KEGG website: https://www.genome.jp/kegg/. ^#^ https://www.gsea-msigdb.org/gsea/msigdb/cards/BIOCARTA_VDR_PATHWAY; ^$^ https://www.gsea-msigdb.org/gsea/msigdb/cards/BIOCARTA_ETC_PATHWAY.

## Supplementary Materials

The following are available online at https://www.mdpi.com/2076-3425/10/8/545/s1, Table S1: Identified proteins with normalized ratio. Table S2: Protein-Peptide Report. Table S3: Differentially expressed proteins identified by the iTRAQ proteomic analysis. Table S4: Pathway analysis was performed using Functional Annotation of DAVID 6.8. Table S5: Primer sequences and length of the amplicon of genes as assayed in RT-qPCR. Figure S1: Pathway diagram of differentially expressed proteins on enriched pathways using DAVID analysis.
