# Supplementary material for: Differential Expression of Multiple Disease-Related Protein Groups Induced by Valproic Acid in Human SH-SY5Y Neuroblastoma Cells
Source: Brain Sci. 2020 Aug 12;10(8):545. doi: 10.3390/brainsci10080545 (PMC7465595; doi:10.3390/brainsci10080545)
Supplement: Supplementary file 1 [file brainsci-10-00545-s001.zip › S1 Fig..pdf]

S1 Fig.

Pathway diagram of differentially expressed proteins on enriched pathways using DAVID analysis. The red star indicates the association between pathway proteins and the differentially expressed proteins we identifed (A) VPA altered the expression of 11 proteins involved in Alzheimer's disease. These include ATP5A1, ATP5B, ATP5D, ATP5F1, ATP5J, SDHA, SDHB, UQCRB, UQCRC1, UQCRC2, and UQCRH. (B) VPA altered the expression of 16 proteins involved in Parkinson's disease. These include ATP5A1, ATP5B, ATP5D, ATP5F1, ATP5J, SDHA, SDHB, SLC25A6, UBA7, UQCRB, UQCRC1, UQCRC2, UQCRFS1, UQCRH, VDAC1, and VDAC3. (C) VPA altered the expression of 15 proteins involved in oxidative phosphorylation. These include ATP5A1, ATP5B, ATP5D, ATP5F1, ATP5J, ATP5L, ATP6V1G1, SDHA, SDHB, UQCRB, UQCRC1, UQCRC2, and UQCRH. (D) VPA altered the expression of 10 proteins involved in ubiquitin mediated proteolysis. These include ANAPC4, CDC23, FBXO2, FBXO4, NEDD4, TRAF6, UBE2D1, UBE2E1, UBE2E3, and UBE2G2. (E) VPA altered the expression of 10 proteins involved in ribosome biogenesis in eukaryotes. These include BMS1, DKC1, GNL2, GTPBP4, LSG1, NOP10, RIOK2, TCOF1, WDR36, and WDR75. Illustration taken from DAVID website (http://david.abcc.ncifcrf.gov/) and KEGG database (http://www.kegg.jp/kegg/kegg1.html). Reprinted with permission from KEGG.

S1 Fig. A

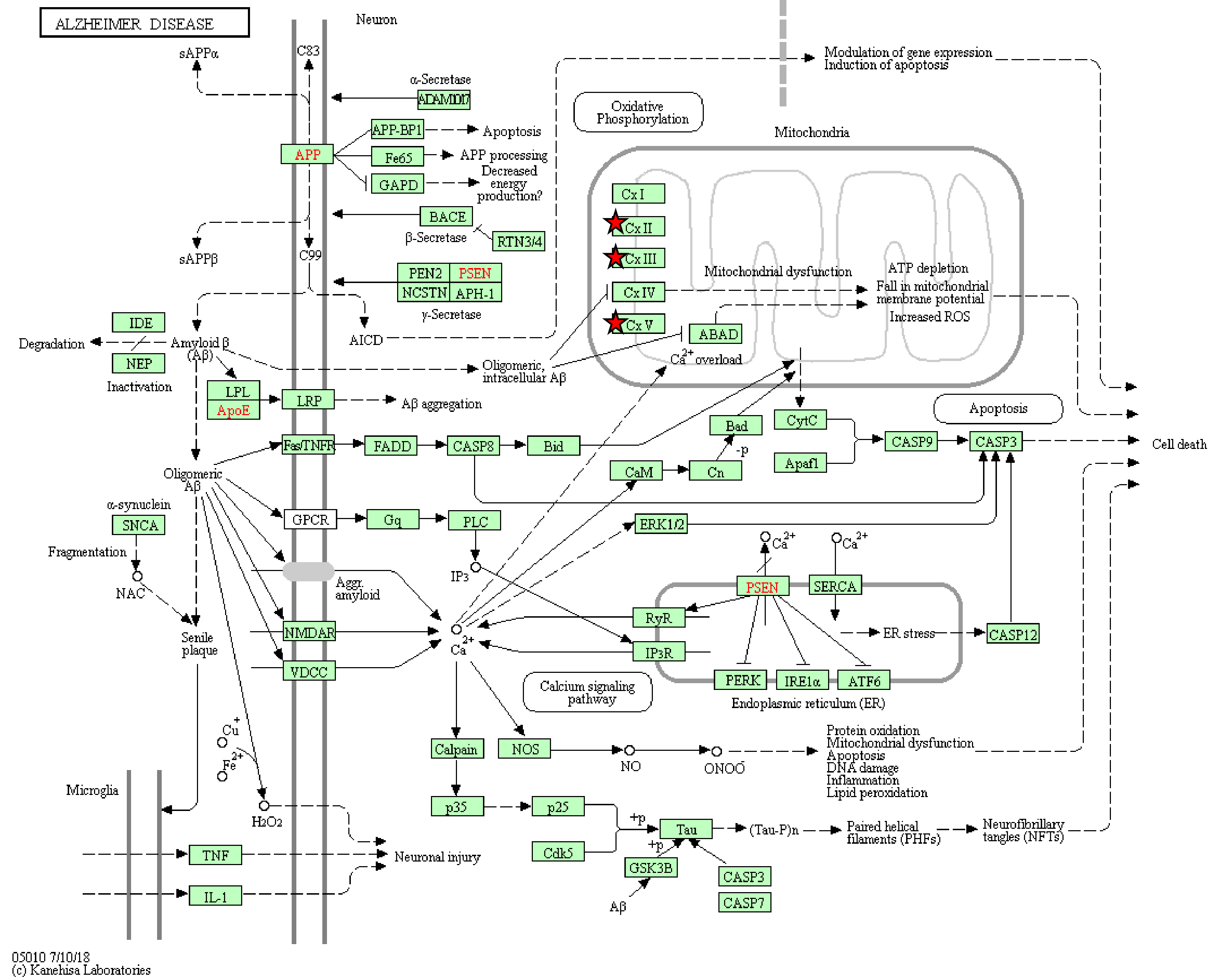
ALZHEIMER DISEASE
Neuron
sAPPα
C83
α-Secretase
ADAM10/17
APP-BP1
Apoptosis
APP
Fe65
APP processing
GAPD
Decreased energy production?
BACE
β-Secretase
RTN3/4
sAPPβ
C99
PEN2
PSEN
NCSTN
APH-1
γ-Secretase
AICD
IDE
NEP
Degradation
Amyloid β (Aβ)
Inactivation
Oligomeric, intracellular Aβ
LPL
ApoE
LRP
Aβ aggregation
Oxidative Phosphorylation
Mitochondria
Cx I
Cx II
Cx III
Cx IV
Cx V
ABAD
Mitochondrial dysfunction
ATP depletion
Fall in mitochondrial membrane potential
Increased ROS
Ca^{2+} overload
Modulation of gene expression
Induction of apoptosis
Apoptosis
Cell death
Fas/TNFR
FADD
CASP8
Bid
Bad
-p
CytC
Apaf1
CASP9
CASP3
CaM
Cn
Oligomeric Aβ
α-synuclein
SNCA
Fragmentation
NAC
Senile plaque
GPCR
Gq
PLC
IP3
ERK1/2
Aggr. amyloid
NMDAR
VDCC
Ca^{2+}
RyR
IP3R
PSEN
SERCA
ER stress
CASP12
PERK
IRE1α
ATF6
Endoplasmic reticulum (ER)
Calcium signaling pathway
Calpain
NOS
NO
ONOO-
Protein oxidation
Mitochondrial dysfunction
Apoptosis
DNA damage
Inflammation
Lipid peroxidation
Cu^{+}
Fe^{2+}
H_2O_2
Microglia
Neuronal injury
TNF
IL-1
p35
p25
Cdk5
+p
Tau
(Tau-P)n
Paired helical filaments (PHFs)
Neurofibrillary tangles (NFTs)
GSK3B
CASP3
CASP7
Aβ
05010 7/10/18
(c) Kanehisa Laboratories

S1 Fig. B

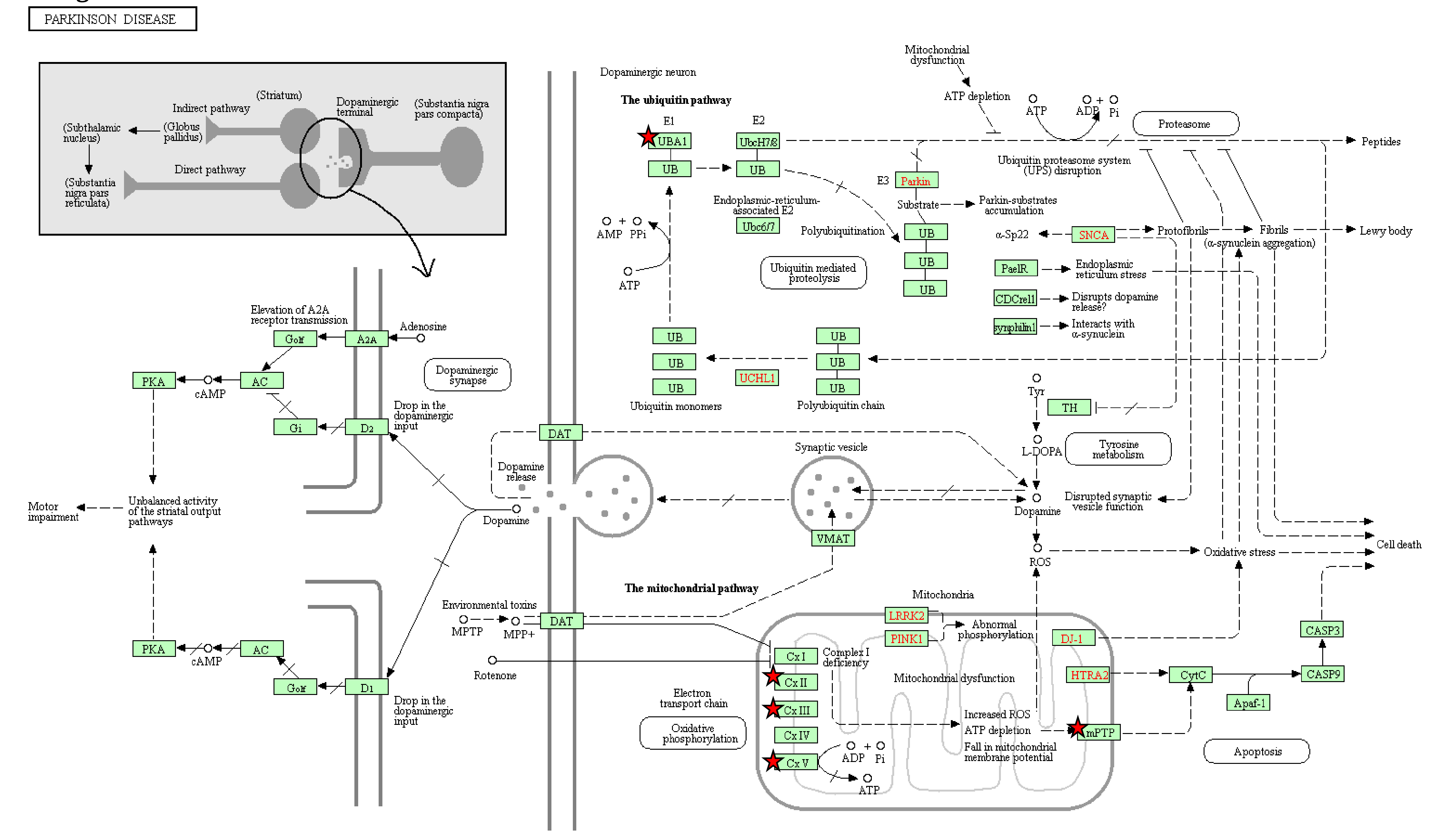

05012 7/10/18

(c) Kanehisa Laboratories

S1 Fig. C

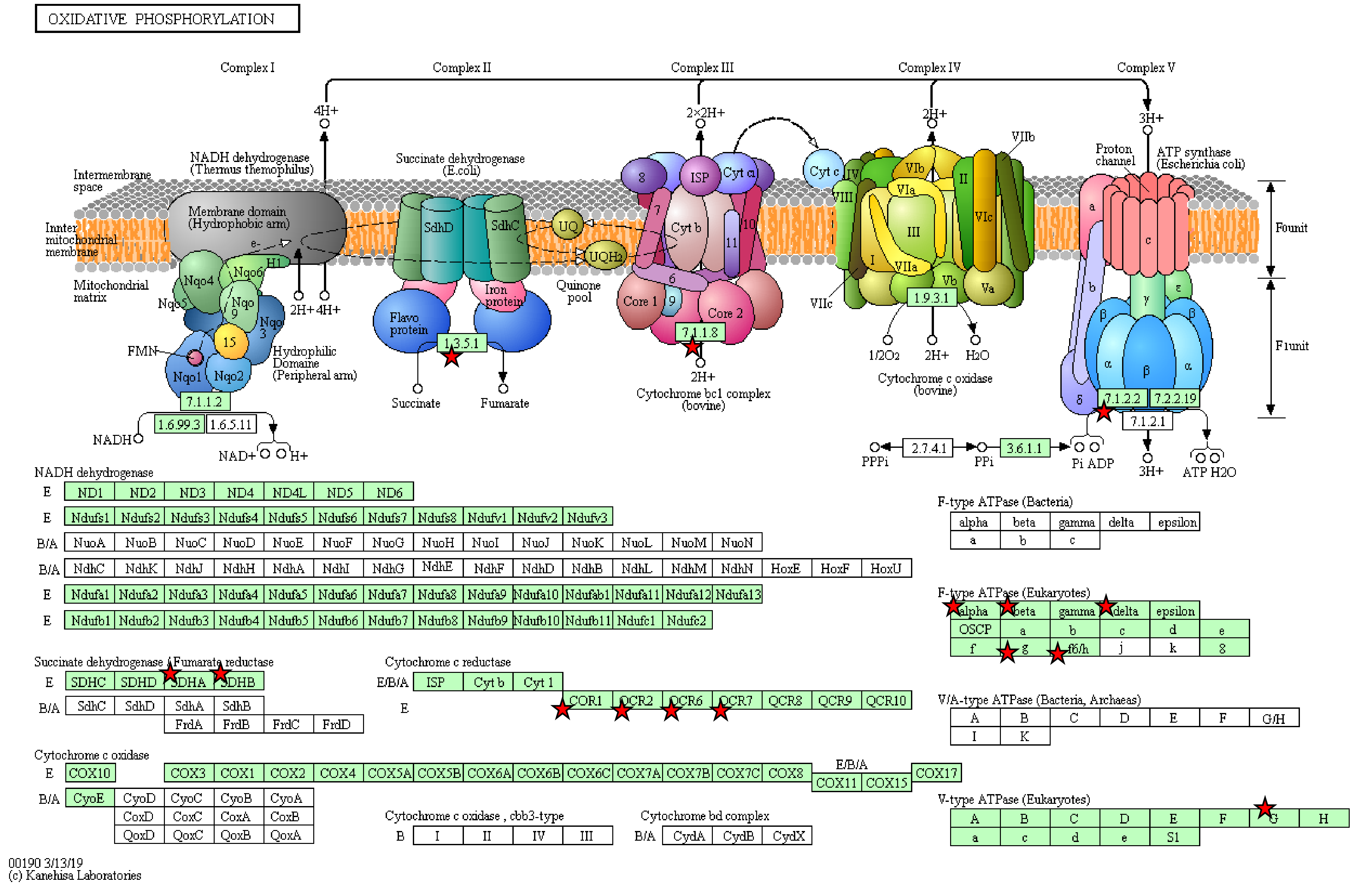
OXIDATIVE PHOSPHORYLATION
Complex I
Complex II
Complex III
Complex IV
Complex V
4H+
2×2H+
2H+
3H+
Intermembrane space
Inner mitochondrial membrane
Mitochondrial matrix
NADH dehydrogenase (Thermus themophilus)
Membrane domain (Hydrophobic arm)
Hydrophilic Domaine (Peripheral arm)
Nqo4
Nqo6
H1
Nqo5
Nqo 9
Nqo 3
15
FMN
Nqo1
Nqo2
2H+ 4H+
7.1.1.2
1.6.99.3
1.6.5.11
NADH
NAD+
H+
Succinate dehydrogenase (E.coli)
SdhD
SdhC
Flavo protein
Iron protein
1.3.5.1
Succinate
Fumarate
UQ
UQH2
Quinone pool
8
ISP
Cyt c1
7
Cyt b
10
11
6
Core 1
9
Core 2
7.1.1.8
2H+
Cytochrome bc1 complex (bovine)
Cyt c
IV
VIII
VIb
VIa
II
III
VIc
I
VIIa
Vb
Va
VIIb
VIIc
1.9.3.1
1/2O2
2H+
H2O
Cytochrome c oxidase (bovine)
Proton channel
ATP synthase (Escherichia coli)
a
c
b
ε
γ
β
α
δ
Fo unit
F1 unit
7.1.2.2
7.2.2.19
7.1.2.1
PPPi
2.7.4.1
PPi
3.6.1.1
Pi ADP
3H+
ATP H2O
NADH dehydrogenase
E ND1 ND2 ND3 ND4 ND4L ND5 ND6
E Ndufs1 Ndufs2 Ndufs3 Ndufs4 Ndufs5 Ndufs6 Ndufs7 Ndufs8 Ndufv1 Ndufv2 Ndufv3
B/A NuoA NuoB NuoC NuoD NuoE NuoF NuoG NuoH NuoI NuoJ NuoK NuoL NuoM NuoN
B/A NdhC NdhK NdhJ NdhH NdhA NdhI NdhG NdhE NdhF NdhD NdhB NdhL NdhM NdhN HoxE HoxF HoxU
E Ndufa1 Ndufa2 Ndufa3 Ndufa4 Ndufa5 Ndufa6 Ndufa7 Ndufa8 Ndufa9 Ndufa10 Ndufab1 Ndufa11 Ndufa12 Ndufa13
E Ndufb1 Ndufb2 Ndufb3 Ndufb4 Ndufb5 Ndufb6 Ndufb7 Ndufb8 Ndufb9 Ndufb10 Ndufb11 Ndufc1 Ndufc2
Succinate dehydrogenase / Fumarate reductase
E SDHC SDHD SDHA SDHB
B/A SdhC SdhD SdhA SdhB
FrdA FrdB FrdC FrdD
Cytochrome c reductase
E/B/A ISP Cyt b Cyt 1
E COR1 QCR2 QCR6 QCR7 QCR8 QCR9 QCR10
Cytochrome c oxidase
E COX10 COX3 COX1 COX2 COX4 COX5A COX5B COX6A COX6B COX6C COX7A COX7B COX7C COX8
E/B/A
COX11 COX15
COX17
B/A CyoE CyoD CyoC CyoB CyoA
CoxD CoxC CoxA CoxB
QoxD QoxC QoxB QoxA
Cytochrome c oxidase , cbb3-type
B I II IV III
Cytochrome bd complex
B/A CydA CydB CydX
F-type ATPase (Bacteria)
alpha beta gamma delta epsilon
a b c
F-type ATPase (Eukaryotes)
alpha beta gamma delta epsilon
OSCP a b c d e
f g f6/h j k 8
V/A-type ATPase (Bacteria, Archaeas)
A B C D E F G/H
I K
V-type ATPase (Eukaryotes)
A B C D E F G H
a c d e S1
00190 3/13/19
(c) Kanehisa Laboratories

S1 Fig. D

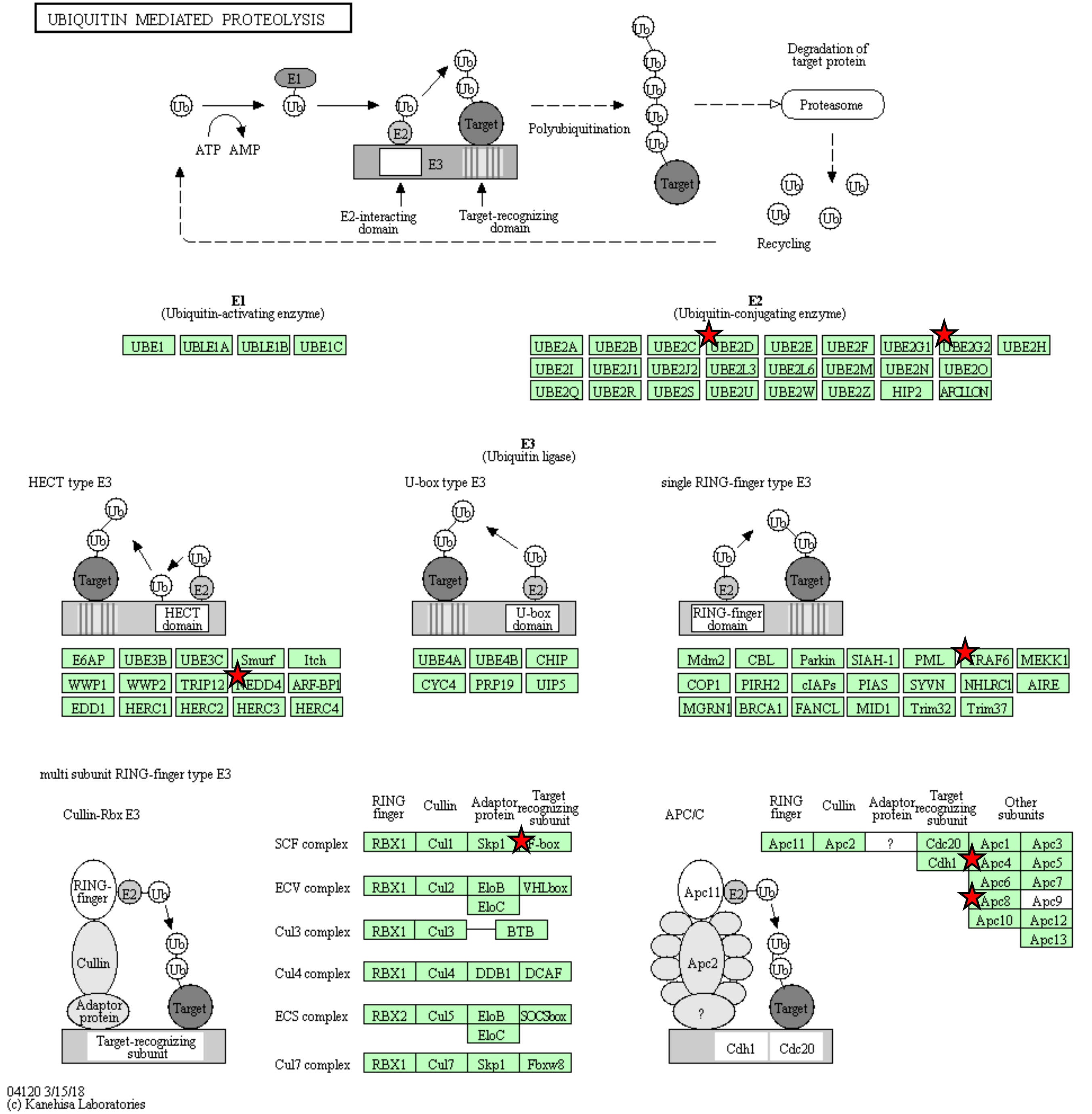
UBIQUITIN MEDIATED PROTEOLYSIS
E1
Ub
ATP AMP
E2
E3
Target
E2-interacting domain
Target-recognizing domain
Polyubiquitination
Degradation of target protein
Proteasome
Recycling
E1
(Ubiquitin-activating enzyme)
UBE1 UBLE1A UBLE1B UBE1C
E2
(Ubiquitin-conjugating enzyme)
UBE2A UBE2B UBE2C UBE2D UBE2E UBE2F UBE2G1 UBE2G2 UBE2H
UBE2I UBE2J1 UBE2J2 UBE2L3 UBE2L6 UBE2M UBE2N UBE2O
UBE2Q UBE2R UBE2S UBE2U UBE2W UBE2Z HIP2 AFCLLON
E3
(Ubiquitin ligase)
HECT type E3
HECT domain
E6AP UBE3B UBE3C Smurf Itch
WWP1 WWP2 TRIP12 NEDD4 ARF-BP1
EDD1 HERC1 HERC2 HERC3 HERC4
U-box type E3
U-box domain
UBE4A UBE4B CHIP
CYC4 PRP19 UIP5
single RING-finger type E3
RING-finger domain
Mdm2 CBL Parkin SIAH-1 PML TRAF6 MEKK1
COP1 PIRH2 cIAPs PIAS SYVN NHLRC1 AIRE
MGRN1 BRCA1 FANCL MID1 Trim32 Trim37
multi subunit RING-finger type E3
Cullin-Rbx E3
RING-finger
Cullin
Adaptor protein
Target-recognizing subunit
RING finger
Cullin
Adaptor protein
Target recognizing subunit
SCF complex RBX1 Cul1 Skp1 F-box
ECV complex RBX1 Cul2 EloB VHLbox EloC
Cul3 complex RBX1 Cul3 BTB
Cul4 complex RBX1 Cul4 DDB1 DCAF
ECS complex RBX2 Cul5 EloB SOCSbox EloC
Cul7 complex RBX1 Cul7 Skp1 Fbxw8
APC/C
RING finger
Cullin
Adaptor protein
Target recognizing subunit
Other subunits
Apc11 Apc2 ? Cdc20 Apc1 Apc3
Cdh1 Apc4 Apc5
Apc6 Apc7
Apc8 Apc9
Apc10 Apc12
Apc13
Apc11
Apc2
?
Cdh1 Cdc20
04120 3/15/18
(c) Kanehisa Laboratories

S1 Fig. E

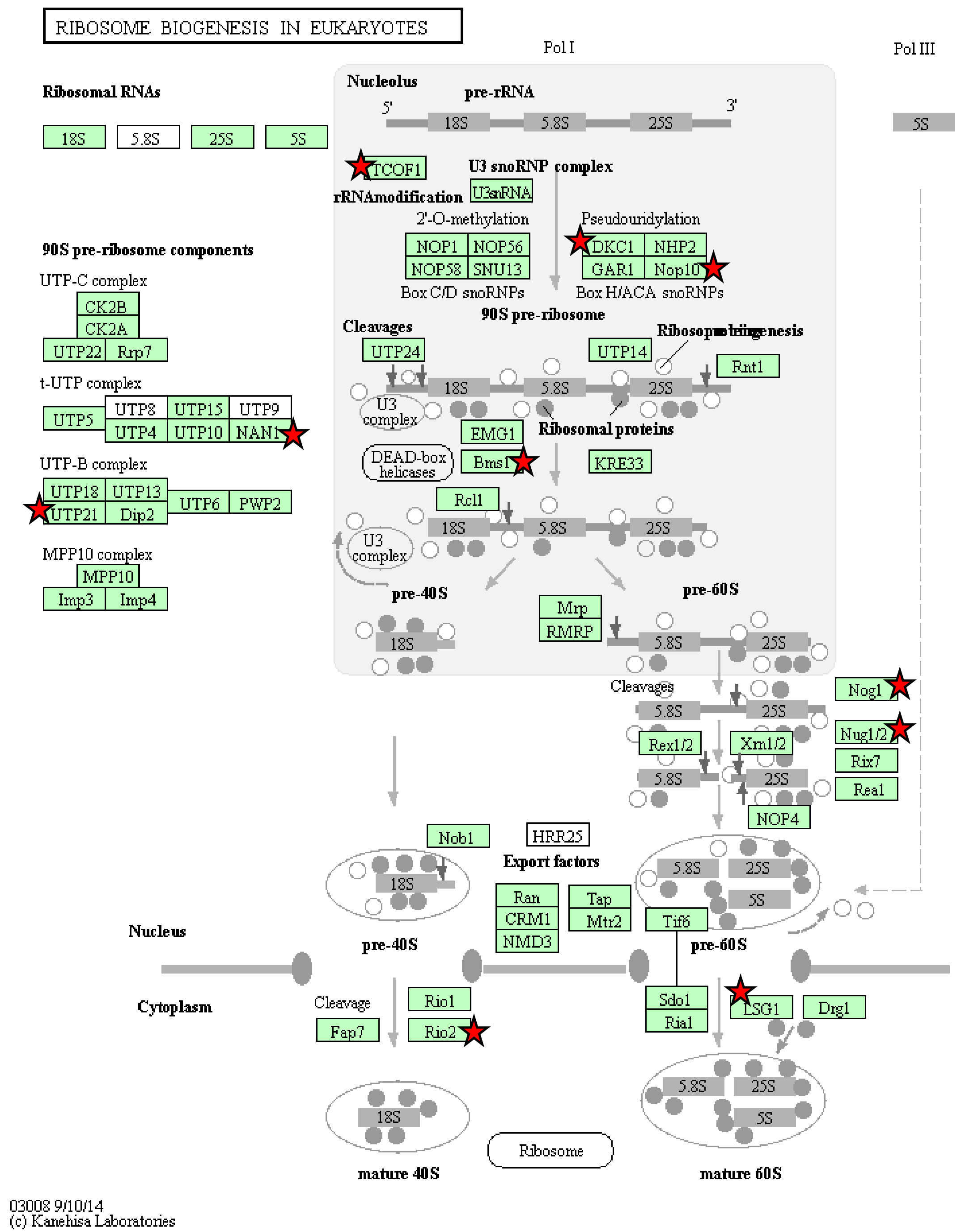
RIBOSOME BIOGENESIS IN EUKARYOTES
Pol I
Pol III
Ribosomal RNAs
18S
5.8S
25S
5S
Nucleolus
pre-rRNA
5'
3'
TCOF1
U3 snoRNP complex
U3snRNA
rRNAmodification
2'-O-methylation
NOP1
NOP56
NOP58
SNU13
Box C/D snoRNPs
Pseudouridylation
DKC1
NHP2
GAR1
Nop10
Box H/ACA snoRNPs
90S pre-ribosome
90S pre-ribosome components
UTP-C complex
CK2B
CK2A
UTP22
Rrp7
t-UTP complex
UTP5
UTP8
UTP15
UTP9
UTP4
UTP10
NAN1
UTP-B complex
UTP18
UTP13
UTP21
Dip2
UTP6
PWP2
MPP10 complex
MPP10
Imp3
Imp4
Cleavages
UTP24
UTP14
Rnt1
U3 complex
EMG1
Ribosomal proteins
DEAD-box helicases
Bms1
KRE33
Rcl1
pre-40S
pre-60S
Mrp
RMRP
Nog1
Nug1/2
Rix7
Rea1
Rex1/2
Xrn1/2
NOP4
Nob1
HRR25
Export factors
Ran
CRM1
NMD3
Tap
Mtr2
Tif6
Nucleus
Cytoplasm
Cleavage
Rio1
Fap7
Rio2
Sdo1
Ria1
LSG1
Drg1
mature 40S
Ribosome
mature 60S
03008 9/10/14
(c) Kanehisa Laboratories
