# Supplementary material for: Differential Expression of Multiple Disease-Related Protein Groups Induced by Valproic Acid in Human SH-SY5Y Neuroblastoma Cells
Source: Brain Sci. 2020 Aug 12;10(8):545. doi: 10.3390/brainsci10080545 (PMC7465595; doi:10.3390/brainsci10080545)
Supplement: Supplementary file 1 [file brainsci-10-00545-s001.zip › S5 Table.doc]

**S5 Table. Primer sequences and length of the amplicon of genes as assayed in RT-qPCR.**

| Genes | Official Full Name | Primer Sequences (5’ to 3’) | | Size (bps) |
| --- | --- | --- | --- | --- |
|  |  | Sense | Antisense |  |
| *ATP5F1A* | ATP synthase F1 subunit alpha | CGACAGACTGGGAAAACCTC | ACTGGGCAACAGTGGATCTC | 136 |
| *ATP5PF* | ATP synthase peripheral stalk subunit F6 | GAGCTGGAGAGGGAGCTTTT | TGGGGTTTTTCGATGACTTC | 113 |
| *BAZ1B* | bromodomain adjacent to zinc finger domain 1B | TACAGTGAGCGCATTTGGAC | CCTCCTTCAAAAGCTCAGCA | 103 |
| *BMS1* | BMS1 ribosome biogenesis factor | AAAAGCGCAGACTCACCATT | AAACCCAAAGCTGGCATCTA | 110 |
| *FBXO2* | F-box protein 2 | TACTTCGCCTCCTCCTTTGA | CCGAGTACCAGTCCTTCACC | 124 |
| *GAPDH* | glyceraldehyde-3-phosphate dehydrogenase | ACCCACTCCTCCACCTTTGA | CTGTTGCTGTAGCCAAATTCGT | 205 |
| *HDAC1* | histone deacetylase 1 | CCATCCGTCCAGATAACATG | AGAACTCAAACAGGCCATCG | 97 |
| *HDAC2* | histone deacetylase 2 | GTTGCTCGATGTTGGACATA | TGGTTTGTCATGTTTGAAGGA | 138 |
| *SDHA* | succinate dehydrogenase complex flavoprotein subunit A | AGGAACCCGAGGTTTTCACT | CCTACCACCACTGCATCAAA | 120 |
| *SMARCA4* | SWI/SNF related, matrix associated, actin dependent regulator of chromatin, subfamily a, member 4 | GGAGTCCATGCATGAGAAGG | GAGGGGTAACCTTGGGAGTG | 138 |
| *UBA7* | ubiquitin like modifier activating enzyme 7 | CTGCTGGCTCATCTTCAGGA | CCTGCTTTTCAGGTGACCAT | 100 |
| *UBE2D1* | ubiquitin conjugating enzyme E2 D1 | CGCTGAAGAGGATTCAGAAAG | CAGGAGGCCCCATAATAGTG | 120 |
| *UQCRB* | ubiquinol-cytochrome c reductase binding protein | ACTGGGGTTAATGCGAGATG | AGTGCCCTCTTAATGCGAAA | 114 |
| *VDAC1* | voltage dependent anion channel 1 | CAAATCTGCCAGGGATGTCT | GTGGTCTCAGTGTTGGCTGA | 123 |
| *WDR36* | WD repeat domain 36 | GGGCAAGAGTTTCCACACCT | ATTACGGGCAAATGCAGAGA | 148 |
